# Comparison of Microwave Sensitivity and Performance of Asphalt Mastic with Various Steel Slag Powders

**DOI:** 10.3390/ma18061348

**Published:** 2025-03-19

**Authors:** Zeyu Geng, Weixiao Yu, Min Jiang, Yinghao Miao

**Affiliations:** 1 National Center for Materials Service Safety, University of Science and Technology Beijing, Beijing 100083, China; m202221253@xs.ustb.edu.cn (Z.G.); yuweixiao@xs.ustb.edu.cn (W.Y.); 2 School of Metallurgical and Ecological Engineering, University of Science and Technology Beijing, Beijing 100083, China; jiangmin@ustb.edu.cn

**Keywords:** road engineering, resource recovery, electromagnetic properties, microwave heating, rheological properties

## Abstract

Steel slag is a common solid waste, but it has good microwave absorbing ability. The poor microwave sensitivity of asphalt mixture limits the development of microwave maintenance for asphalt pavement. Therefore, it is significant to apply steel slag to asphalt pavement. This study analyzes the difference in the microwave sensitivity and performance between the asphalt mastics with blast furnace slag powder (BFSP), converter slag powder (CSP), refined slag powder (RSP), and limestone powder (LP). First, the chemical composition of BFSP, CSP, RSP, and LP is analyzed by X-ray diffractometer (XRD) and X-ray fluorescence (XRF) tests. Then, the micromorphology characteristics of the asphalt mastic with BFSP, that with CSP, that with RSP, and that with LP are studied using atomic force microscope (AFM) tests. Finally, the rheological properties of the four asphalt mastics are investigated through dynamic shear rheometer (DSR) and bending beam rheometer (BBR) tests. The results show that steel slag powder can effectively improve the microwave sensitivity of asphalt mastic. RSP and CSP can improve the anti-deformation ability of asphalt mastic. In addition, steel slag powders have an adverse effect on the low-temperature cracking resistance of asphalt mastic, but the creep strength and creep rate of asphalt mastic with steel slag powder are within a reasonable range. In general, steel slag powder as filler has great application potential in road engineering. However, it has a certain influence on the performance of asphalt mastic. It is necessary to carry out targeted selection in practical engineering.

## 1. Introduction

Steel slag is a solid waste in the production process of iron and steel. According to the treatment technology, it can be divided into blast furnace slag (BFS), converter slag (CS), refining slag (RS), and so on. BFS is the solid waste discharged during the smelting of pig iron in the blast furnace, CS is the main production of steelmaking, and RS is the solid waste in the secondary metallurgical process. The treatment method of steel slag has the disadvantages of the incomplete separation between slag and iron and the low grade of recovered scrap [1,2]. Approximately 12–20 tons of steel slags are manufactured when 100 tons of steels are produced [3,4]. The China Statistical Yearbook 2022 shows that about 1.035 billion tons of crude steel are produced in 2022, which means that about 155–207 million tons of steel slags are generated. A large amount of steel slag is piled up, not only occupying a large space but also producing lots of environment-polluted dust. Therefore, it is necessary to explore the utilization method of steel slags as resources to reduce environmental pollution.

Due to the unique morphological characteristics and physical properties, steel slag has been widely used in the performance optimization research of asphalt mixtures. The surface pores of steel slag can enhance the adhesion between steel slag and asphalt through adsorption, which improves resistance to external loads [5]. Compared with conventional aggregates, steel slag aggregates have a lower wear rate, higher initial angularity, and better wear resistance [6]. These characteristics can improve the skeleton quality and the low-temperature crack resistance of asphalt mixtures [7,8]. However, steel slag has volume instability. The f-CaO and f-MgO in steel slag undergo hydration reactions with water, causing the steel slag to expand in a humid environment and the asphalt mixture to crack [9].

To eliminate the volume expansion problem of steel slag asphalt mixtures, Wu et al. [10] and Chen et al. [11] used steel slag powder as the filler. The results showed that asphalt mixtures with steel slag powder not only have good volume stability but also excellent performance in terms of rutting resistance. Based on the high correlation between the performance of asphalt mastic and that of asphalt mixtures, some researchers have evaluated the rheological properties of various steel slag powder asphalt mastics. Lagos-Varas et al. [12] studied the rheological properties of asphalt mastics with hydrated ladle slag (i.e., refining slag) at different dosages and found that a filler-to-asphalt ratio of 0.75 has the best improvement effect on the rheological properties of the asphalt mastic. Li et al. [13] indicated that alkaline oxygen furnace slag (i.e., converter slag) powder was a potential substitute for limestone powder, and the alkaline oxygen furnace slag powder obtained by grinding steel slag with a size of 0–9.5 mm had the best modification effect on asphalt. Wang et al. [14] found that both thin-film oven aging and ultraviolet aging can increase the stiffness and fatigue life of the asphalt mastic, and the fatigue life of the alkaline oxygen furnace slag asphalt mastic after ultraviolet aging was better than that of the limestone powder asphalt mastic. It can be seen that steel slag powder is a potential substitute for limestone mineral powder.

Microwave heating technology is an economical and environmentally friendly way of in-situ rehabilitation [15], which enables in-situ heat recovery, repair of crack damage in asphalt pavements, and de-icing of pavements during winter maintenance operations [16]. It has high requirements for the microwave sensitivity of materials [17]. The poor microwave absorption performance of asphalt mixture limits the development of microwave heating technology in road engineering [18,19]. Steel slag contains abundant metal oxides, resulting in good microwave-absorbing properties. Chen et al. [20] studied the temperature distribution characteristics of steel slag aggregate asphalt mixtures under microwave heating and found that the average heating rates of the surface and interior of steel slag porous asphalt concrete were higher than those of basalt porous asphalt concrete. Liu et al. [21] showed that the microwave heating uniformity of asphalt mixtures was best when the percentage of steel slag in the aggregate was less than 75%, but multiple microwave heating cycles led to an increase in the proportion of large and oversized voids within the mixtures, which caused the decrease in the durability. In contrast, Wang et al. [22] found that microwave heating was effective in rehabilitating steel slag asphalt mixtures of dense-graded and interrupted-graded mixtures by restoring the porosity level to the initial state. Luo et al. [23] found that the thermal field distribution of asphalt mixtures was the most uniform at 2.45 GHz, but local overheating still occurred. Wang et al. [24] found that alkaline oxide furnace slag filler had excellent electromagnetic properties and was able to improve the microwave heating efficiency of asphalt mastic, which was suitable for the construction of functional pavements with microwave rapid repair performance. In summary, steel slag aggregate performs well in improving the microwave heating efficiency of asphalt mixtures but can adversely affect the heated asphalt mixtures. Steel slag powder not only improves the performance of asphalt mastic but also enhances the microwave sensitivity of asphalt mastic.

However, based on the different steelmaking processes and raw materials, there are differences in the chemical and physical properties of different steel slags, so it is necessary to compare and analyze the effect of different steel slag powders on the road performance and microwave sensitivity of asphalt mastic. In this study, BFSP, CSP, and RSP are used as research objects, and their chemical compositions and electromagnetic properties are compared to explore their microwave sensitivity enhancement effects on asphalt. Meanwhile, the effect of road performance of asphalt mastics with different steel slag powders is analyzed.

## 2. Materials and Methods

### 2.1. Materials

#### 2.1.1. Steel Slag and Limestone Powders

**Figure 1:**
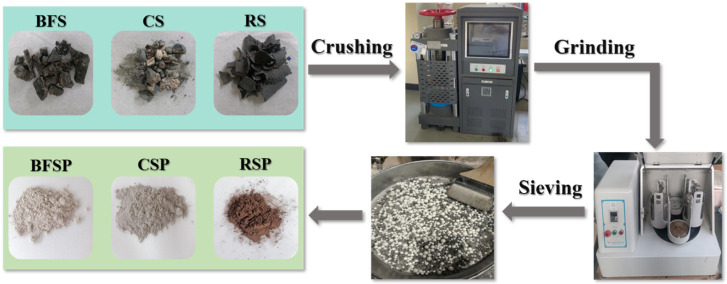
Preparation process of three steel slag fillers.

BFSP, CSP, and RSP used in this study were supplied by Zenith Steel Group Co., Ltd. (Changzhou, China), and LP was supplied by Beijing Municipal Road & Bridge Building Material Group Co., Ltd. (Beijing, China). BFSP, CSP, and RSP were selected as fillers to prepare microwave sensitivity-enhanced asphalt mastic, and LP was selected as filler to prepare common asphalt mastic to be used as the control group. The preparation process of steel slag fillers was as follows: (1) crushing the large-sized steel slags into small particles by a press machine, (2) grinding the small steel slag particles into powders using the planetary ball mill, and (3) sieving the steel slag powders using a 0.075 mm sieve. Figure 1 depicts the preparation process of three steel slag fillers. The technical properties of steel slag and limestone fillers (apparent density, hydrophilic coefficient, and heat invariability) were measured according to Chinese Technical Specification Test Methods of Aggregates for Highway Engineering (JTG 3432-2024 [25]), as shown in Table 1. As can be seen from Table 1, the hydrophilic coefficient of CSP was larger than 1, indicating that CSP was more likely to swell in water. The hydrophilic coefficients of BFSP, RSP, and LP were smaller than 1, indicating that they had a stronger affinity to asphalt than water.

#### 2.1.2. Asphalt

Xinhai 70# asphalt was selected in this study. Its technical indicators are listed in Table 2.

### 2.2. Preparation of Asphalt Mastics with Different Fillers

**Figure 2:**
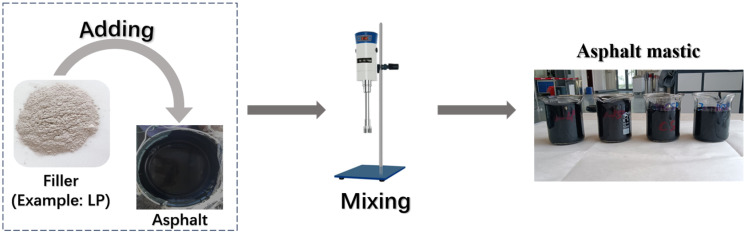
Preparation process of asphalt mastic.

All fillers were dried in an oven for 24 h at 105 °C before preparing asphalt mastic. The mass ratio of limestone filler to asphalt was 1:1, and that of each steel slag filler to asphalt, as shown in Table 3, was determined by replacing limestone filler with an equal volume of steel slag filler. The asphalt and filler were stirred for 45 min with the speed of 2500 r/min using a digital high-speed mixer at 135–150 °C. During the mixing process, the filler was added into asphalt multiple times. Figure 2 describes the preparation process of asphalt mastic. For convenience, 70-LP was employed to represent the asphalt mastic with limestone filler, and the same pattern was used to denote the asphalt mastics with steel slag fillers.

### 2.3. Test Methods for Properties of Steel Slag and Limestone Powders

The electromagnetic performance of steel slag and limestone powders is closely related to their chemical composition, and it determines the microwave sensitivity of corresponding asphalt mastics.

The difference in the chemical composition between BFSP, CSP, RSP, and LP contributes to understanding the microwave sensitivity-enhanced mechanism of steel slag powders. Therefore, the X-Ray Diffractometer (XRD, D8 Advance, Bruker, Rheinstetten, Germany)test. X-Ray Fluorescence (XRF, Zetium, Malvern Panalytical, Malvern, UK) text, and electromagnetic performance tests were conducted on steel slag and limestone powders.

#### 2.3.1. XRD Test

An XRD test was conducted on steel slag and limestone powders to analyze the internal material parameters such as the number of atoms in the crystal lattice and crystal size. Analyses were performed using Cu targets and reflection patterns. Samples were prepared using the tablet method. The diffraction angle was 5–90°, the scan frequency was 2°/min, the scan step was 0.02°, and the angular accuracy was 0.0001°.

#### 2.3.2. XRF Test

An XRF test was conducted on steel slag and limestone powders adopting the press-flake method to quantitatively analyze the oxide composition. The samples were prepared by the tablet method. The elements were analyzed in the range of 9F to 92U.

#### 2.3.3. Electromagnetic Performance Test

The electromagnetic parameters of BFSP, CSP, RSP, and LP were measured using a Vector Network Analyzer through the coaxial method in the frequency range of 2 GHz to 18 GHz to evaluate the microwave absorption ability of four fillers. The weight ratio of filler to paraffin wax is 4:1. The inner diameter, external diameter, and height of the coaxial ring were 3.04 mm, 7 mm, and 2 mm, respectively.

### 2.4. Test Methods for Performance of Asphalt Mastics

The microwave heating tests were conducted on 70-BFSP, 70-RSP, 70-CSP, and 70-LP to determine the microwave sensitivity improvement in asphalt mastics by steel slag powders. The steel slag powders can be used as fillers under the condition that the performances of asphalt mastics meet requirements, so dynamic shear rheometer (DSR) and bending beam rheometer (BBR) tests are conducted to understand the performances of asphalt mastic. The atomic force microscope (AFM) test is carried out to explain the influence mechanism of steel slag powder on the performance of asphalt mastic.

#### 2.4.1. Microwave Heating Test

A microwave heating test was carried out using an FCMCR-3C type microwave reactor to analyze the microwave sensitivity of the asphalt mastics with different fillers. The initial temperature and volume of each asphalt mastic were, respectively, 25 °C and 25 mL. The heating frequency of the microwave reactor was 2.45 GHz, and its output power was 300 W. The heating time was 5 min. The temperature of asphalt mastic was recorded per second using a Teflon-coated Pt100 platinum resistance temperature sensor (Kerui Instrument Co., Gongyi, China).

#### 2.4.2. DSR Test

The Smart Pave 102 type DSR equipment was applied in this study to investigate the high-temperature rheological properties of asphalt mastic. The frequency sweep test was conducted in strain-controlled mode to evaluate the viscoelasticity and anti-rutting performance of different asphalt mastics. The sweep frequency range was from 0.01 to 100 rad/s, and the sweep temperature was, respectively, 15 °C, 25 °C, 35 °C, 45 °C, 55 °C, 65 °C, and 75 °C.

The multiple stress creep recovery (MSCR) test was carried out to assess the permanent deformation resistance of different asphalt mastics. According to AASHTO T350-19 [26], two constant stresses of 0.1 kPa and 3.2 kPa and the temperature of 60 °C were selected.

#### 2.4.3. BBR Test

The BBR test was performed according to AASHTO T313-19 [27] to evaluate the low-temperature stiffness and stress relaxation characteristics of asphalt mastics with different fillers. The test was conducted under 980 mN for 240 s. The test temperature was −12 °C.

#### 2.4.4. AFM Test

An AFM test was carried out at the test temperature of 25 °C using a Dimension Icon AFM device to intuitively observe the difference in the microstructure between asphalt mastics with different fillers. The surface morphology of asphalt mastic is acquired by the Peak Force QNM model with a scanning range of 20 μm × 20 μm.

## 3. Properties of Steel Slag and Limestone Powders

### 3.1. Chemical Composition

**Figure 3:**
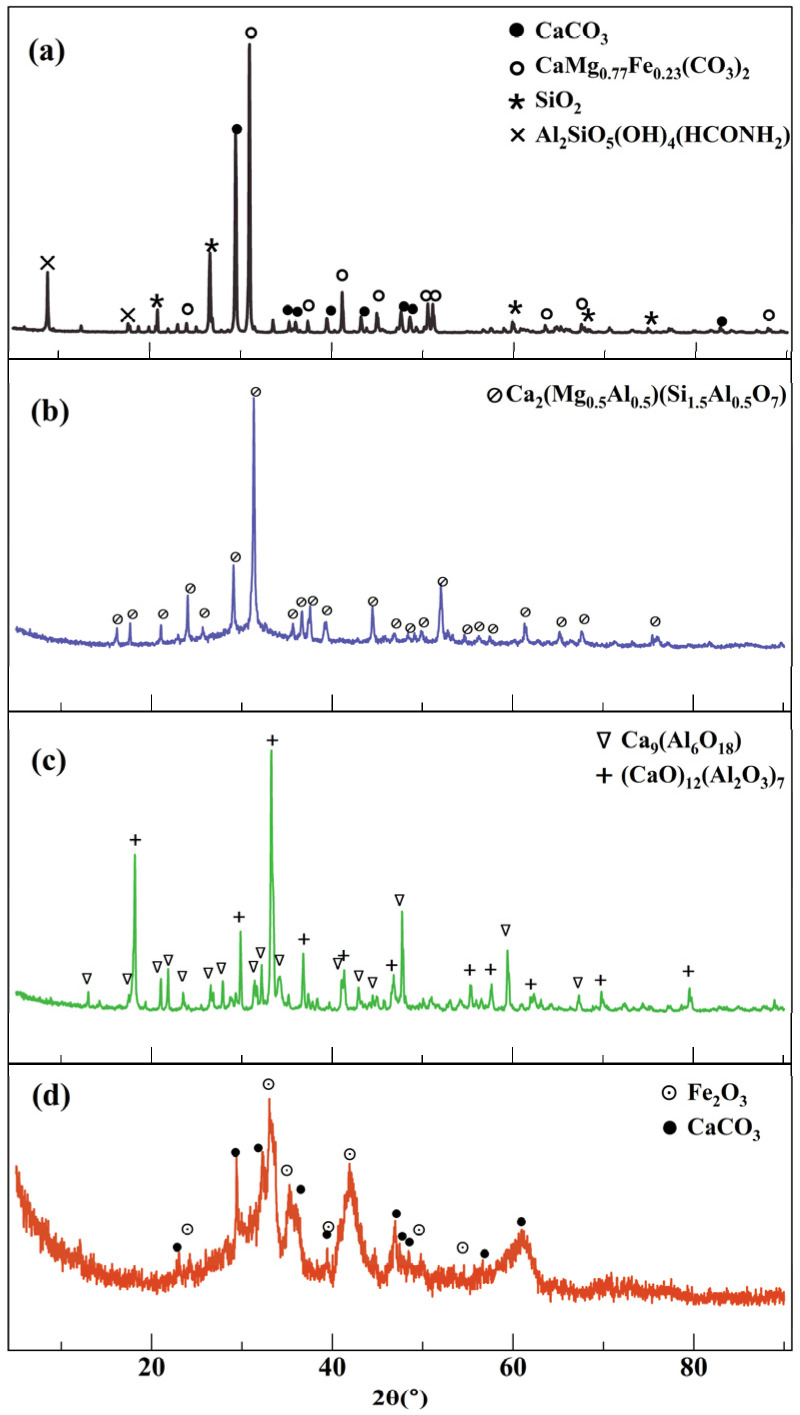
XRD test results of different powders: (**a**) LP, (**b**) BFSP, (**c**) CSP, and (**d**) RSP.

The chemical compositions of LP, BFSP, CSP, and RSP are analyzed to understand the influence mechanism of steel slag and limestone powders on the microwave sensitivity of asphalt mastic. Figure 3 depicts the phase compositions of the four powders. As can be seen, the peak intensities of the phases of the four powders are pronounced, indicating they have a high purity degree. The primary mineral phase of LP is calcium carbonate and dolomite, that of BFSP is akermanite–gehlenite, that of CSP is calcium aluminum. RSP is mainly composed of iron oxide and calcite.

Table 4 shows the chemical composition of LP, RSP, CSP, and BFSP. It can be seen that the content of CaO is highest and that of Fe_2_O_3_ is smallest in LP. BFSP contains a high content of SiO_2_ and CaO and a low content of Fe_2_O_3_. CSP is mainly composed of CaO and Al_2_O_3_. The chemical compositions of RSP mainly are CaO, Fe_2_O_3_, and SiO_2_. The XRF characterization also further verifies the reliability of the XRD results.

### 3.2. Microwave Absorption Property

**Figure 4:**
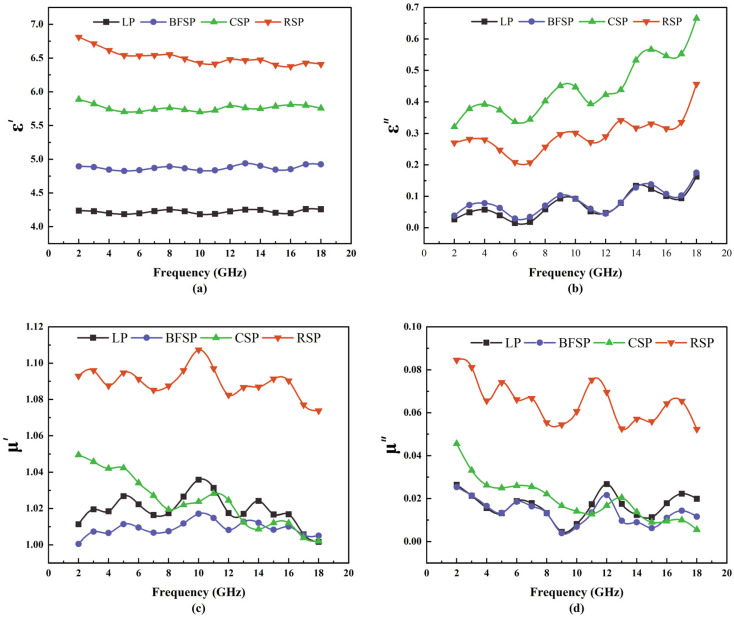
Electromagnetic parameters of four fillers: (**a**) ε′, (**b**) ε″, (**c**) μ′, and (**d**) μ″.

The microwave absorption ability is related to the electromagnetic parameters of materials, which include relative complex permittivity (ε_r_ = ε′ − jε″) and relative magnetic permeability (μ_r_ = μ′ − jμ″). The real parts ε′ and μ′ denote the microwave energy storage capacity of materials. The imaginary parts ε″ and μ″ reflect the microwave energy loss ability of materials. Figure 4 presents the electromagnetic parameters of LP, BFSP, CSP, and RSP. As can be seen, with the increase in frequency, ε′ remains basically unchanged, ε″ shows a rising tendency, and μ′ and μ″ show a downward trend. CSP and RSP have higher values of ε′ and ε″ compared with LP and BFSP. RSP has the largest values of μ′ and μ″. In a low-frequency range (2–8 GHz), the μ′ and μ″ of CSP are higher than those of LP and BFSP. In a high-frequency range (12–18 GHz), there is no significant difference in the μ′ and μ″ between CSP, LP, and BFSP. The above results show that RSP is a dielectric and magnetic loss microwave-absorbing material. CSP is a dielectric loss microwave absorbing material. BFSP has better dielectric loss capability. LP has worse electromagnetic performance. Steel slag powders are more sensitive to microwave radiation than LP.

The principle of microwave absorption is to convert the microwave energy into thermal energy through a physical mechanism and the dissipation of the motion. Magnetic and dielectric losses are two main microwave energy conversion mechanisms, which are, respectively, denoted as the angular tangent of the dielectric loss (tan*δ_ε_* = ε″/ε′) and the angular tangent of the magnetic loss (tan*δ_μ_* = μ″/μ′). Higher loss angle tangent represents that the material could convert microwave energy into more thermal energy through the corresponding loss mechanism. Therefore, the loss angle tangent (tan*δ*) is closely related to microwave absorption ability, which can be calculated by Equation (1). The larger the loss angle tangent, the better the microwave absorption ability.

(1) $$\tan\delta =\tan\delta_{\varepsilon }+\tan\delta_{\mu }$$

**Figure 5:**
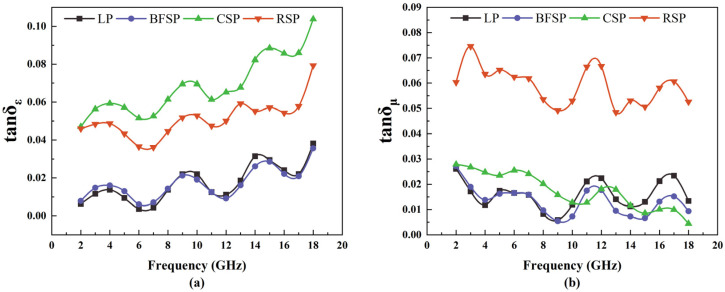
Magnetic and dielectric losses of four fillers: (**a**) tan*δ_ε_* and (**b**) tan*δ_μ_*.

Figure 5 depicts tan*δ_ε_* and tanδ_μ_ of LP, BFSP, CSP, and RSP. As can be seen, with the increase in frequency, tan*δ_ε_* of four fillers shows a rising tendency, and tan*δ_μ_* of four fillers shows a downward trend. RSP has higher values of tan*δ_ε_* and tan*δ_μ_.* CSP has a higher value of tan*δ_ε_* and a lower value of tan*δ_μ_.* Both tan*δ_ε_* and tan*δ_μ_* of LP and BFSP are relatively small. The above results show that the synergistic effect of dielectric and magnetic loss of RSP is best in the four fillers, indicating that RSP has the strongest ability to convert the microwave energy into thermal energy. CSP has a good dielectric loss ability, but its magnetic loss ability is poor. Both LP and BFSP have worse dielectric and magnetic loss properties. However, the dielectric loss ability of BFSP is higher than that of LP in the low-frequency range.

**Figure 6:**
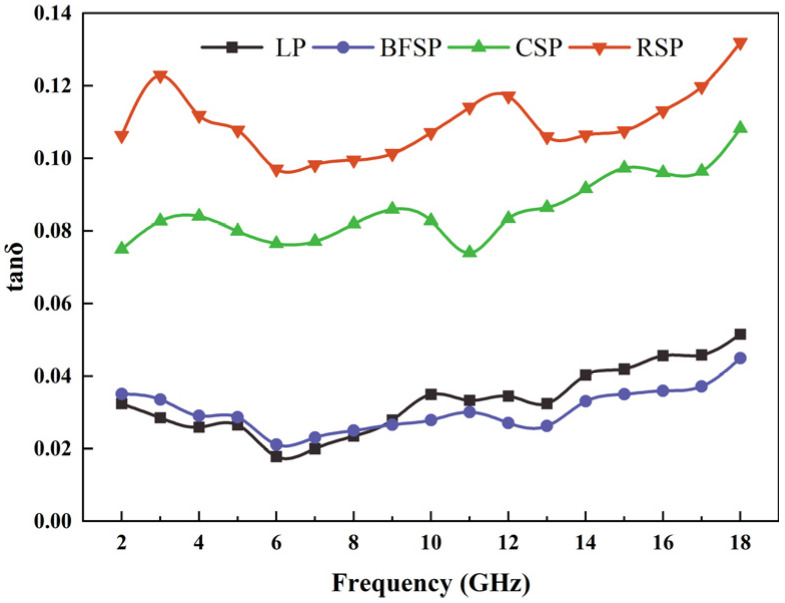
Loss angle tangent of fillers.

Figure 6 shows the microwave absorption ability of LP, BFSP, CSP, and RSP. As can be seen, RSP has the strongest microwave absorption performance, followed by CSP. The microwave absorption ability of BFSP is better than that of LP at 2–8 GHz, while the result is opposite at 10–18 GHz. This is because the magnetic material Fe_2_O_3_ plays a leading role in the microwave absorption ability, and its content in RSP is far higher than that in the other three fillers. In addition, the dielectric material Al_2_O_3_ also has a certain influence on the microwave absorption ability, and its content in the fillers follows the order of CSP > BFSP > LP.

## 4. Microwave Sensitivity of Asphalt Mastics with Different Fillers

### 4.1. Heating Curve

**Figure 7:**
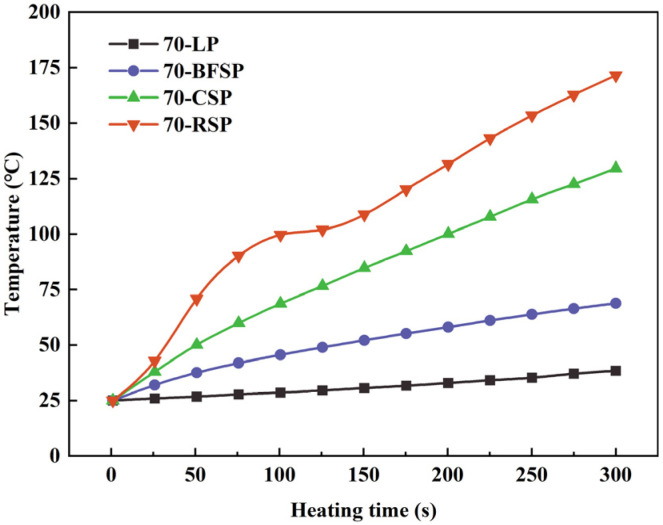
Heating curves of asphalt mastics with different fillers.

Figure 7 describes the heating curves of asphalt mastics with different fillers. It can be seen that the temperature of each asphalt mastic gradually rises as microwave heating time increases. After microwave heating for 300 s, the temperature of 70-LP, 70-BFSP, 70-CSP, and 70-RSP reaches 38 °C, 69 °C, 130 °C, and 172 °C, respectively. Under the same heating time, the temperature of the asphalt mastic with steel slag filler is higher than that of asphalt mastic with limestone filler, indicating that the addition of steel slag powder achieves a large amount of heat formation in a short time and improves the microwave sensitivity of asphalt mastic. RSP has the best effect to enhance the microwave absorption of asphalt mastic, followed by CSP and BFSP.

The heating curves are fitted to understand the relationship between temperature and microwave heating time for asphalt mastics. Table 5 lists the fitting results. As can be seen, there is a good linear relation between the heating time and temperature, with correlation coefficients of above 0.96. The slopes of fitting equations for asphalt mastics with different fillers follow the order of 70-RSP > 70-CSP > 70-BFSP > 70-LP.

### 4.2. Heating Rate

**Figure 8:**
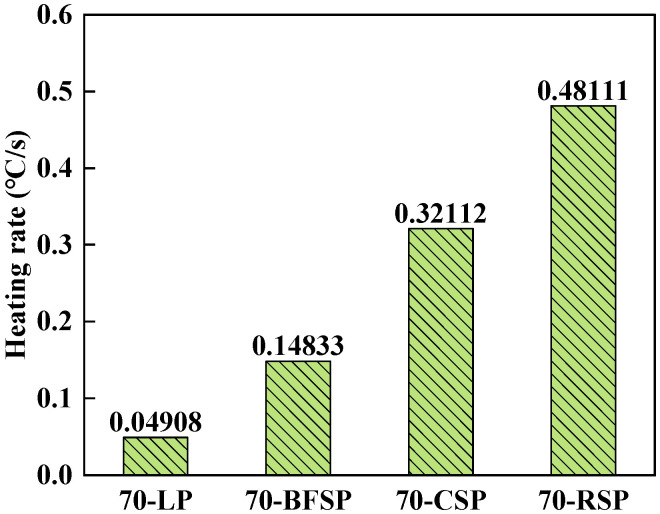
Heating rate of asphalt mastics with different fillers.

The ratio of the temperature difference between 0 s and 300 s to the heating time is defined as the heating rate to analyze the response effect of asphalt mastics with different fillers to microwave heating. Figure 8 depicts the heating rate of each asphalt mastic. It can be seen that the microwave heating rate of asphalt mastics follows the order of 70-RSP > 70-CSP > 70-BFSP > 70-LP. The heating rate of 70-BFSP, 70-CSP, and 70-RSP is, respectively, 3.02, 6.51, and 9.80 times that of 70-LP. The results further proved that the addition of steel slag powder effectively improves the microwave absorption ability of asphalt mastics.

**Figure 9:**
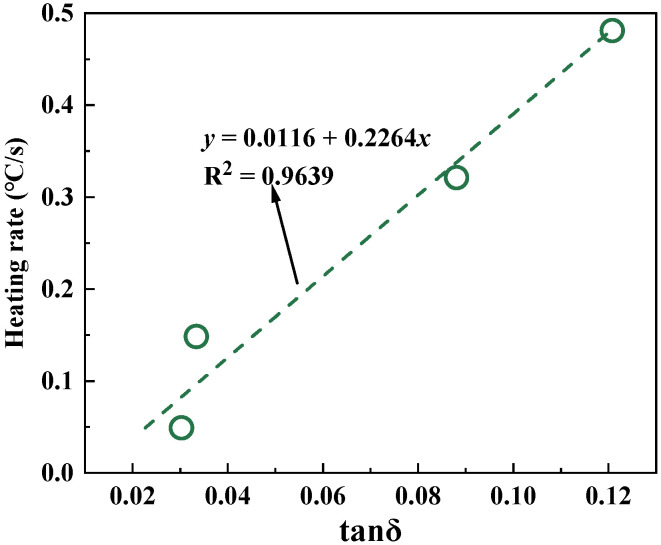
Correlation between the electromagnetic property of fillers and the heating rate of corresponding asphalt mastics.

Figure 9 shows the correlation between the electromagnetic property of fillers and the heating rate of corresponding asphalt mastics at 2.45 GHz. As can be seen, there is a good linear relationship between the electromagnetic property of fillers and the microwave sensitivity of corresponding asphalt mastics, with correlation coefficients of above 0.96.

## 5. Performance of Asphalt Mastics with Different Fillers

The above results show that the addition of steel slag powder can effectively improve the microwave sensitivity of asphalt mastics. However, for realizing its application on the microwave maintenance of asphalt pavement, the performance of asphalt mastic with steel slag filler needs to be further investigated.

### 5.1. Viscoelastic Characteristics

**Figure 10:**
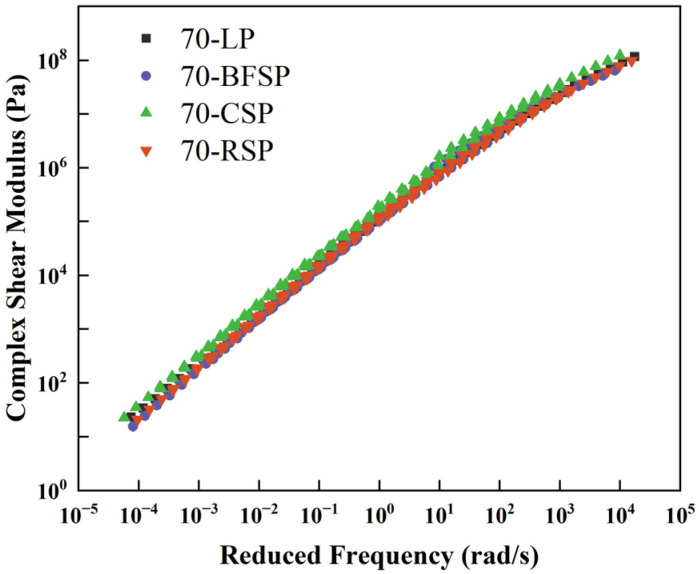
Master curves of complex shear modulus.

**Figure 11:**
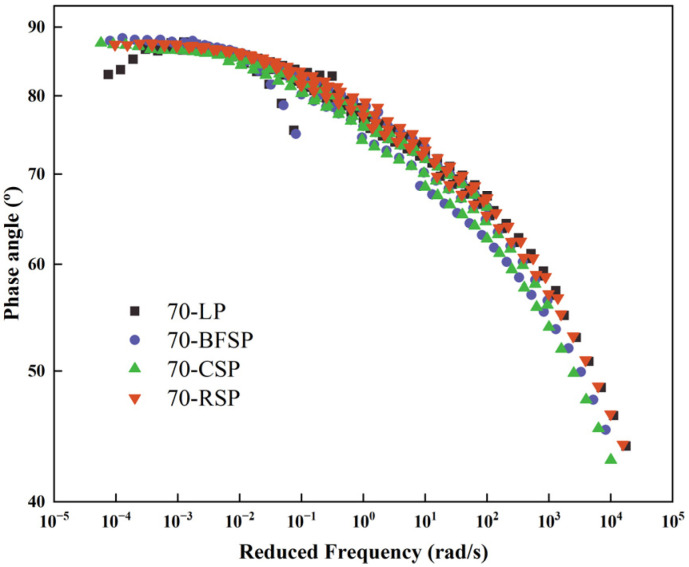
Master curves of phase angles.

According to the frequency sweep test, the complex shear modulus (G*) and phase angle (δ) are two important parameters evaluating the viscoelastic characteristics of asphalt mastics. The composite shear modulus is the ratio of the maximum shear stress to the corresponding shear strain. It reflects the ability to resist the deformation of asphalt mastic. The phase angle is the ratio of the loss modulus to the storage modulus, and it indicates the components of viscosity and elasticity of asphalt mastic. The larger the storage modulus, the better the ability to recover from deformation. The larger the loss modulus, the better the ability to resist deformation. Therefore, the asphalt mastic with the smaller phase angle has better elastic properties. According to the frequency sweep results and time-temperature superposition principle, the master curves of complex shear modulus and phase angle at the reference temperature of 35 °C are constructed based on the CAM model to analyze the viscoelasticity of asphalt mastics, as shown in Figure 10 and Figure 11.

As shown in Figure 10, there is no significant difference in the master curves of complex shear modulus between 70-LP, 70-BFSP, and 70-RSP, indicating that BFSP and RSP have little influence on the stiffness of asphalt mastic. Moreover, 70-CSP has the largest complex shear modulus, indicating that CSP improves the stiffness of asphalt mastic. As can be seen from Figure 11, in the frequency range of 10^−3^–10^−2^ rad/s, there is no significant difference in the phase angles between 70-LP, 70-BFSP, 70-CSP, and 70-RSP. When the frequency is greater than 0.1 rad/s, 70-BFSP and 70-CSP have relatively small phase angles compared with 70-LP and 70-RSP. The above results indicated that RSP would not destroy the elastic properties of asphalt mastic, and BFSP and CSP can improve the elastic properties of asphalt mastic.

### 5.2. Resistance to Rutting

**Figure 12:**
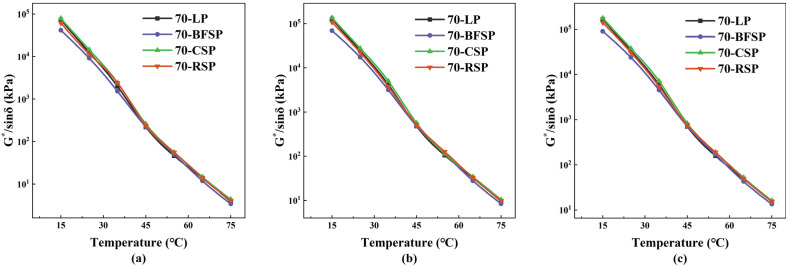
Change in G*/sinδ with temperature at different frequencies: (**a**) 25.1 rad/s, (**b**) 63.1 rad/s, and (**c**) 100 rad/s.

The rutting factor (G*/sinδ) is a key indicator for assessing the rutting resistance of asphalt mastic. The larger the G*/sinδ, the stronger the resistance to rutting deformation. The loading frequency of the DSR test has a corresponding relationship with the travelling speed. The loading frequency of 5 Hz corresponds to a travelling speed of about 30–40 km/h, and that of 10 Hz corresponds to a travelling speed of about 60–65 km/h [28]. On urban roads, the travelling speed of vehicles is between about 30 km/h and 80 km/h. Therefore, the changes of G*/sinδ with temperature at 100 rad/s (16 Hz), 63.1 rad/s (10 Hz), and 25.1 rad/s (4 Hz) are analyzed, as shown in Figure 12. It can be seen that the 70-BFSP has the smallest G*/sinδ, indicating that BFSP decreases the rutting resistance of asphalt mastic. There is no significant difference in the G*/sinδ between 70-CSP, 70-RSP, and 70-LP, indicating that CSP and RSP have little effect on the rutting resistance of asphalt mastic.

**Figure 13:**
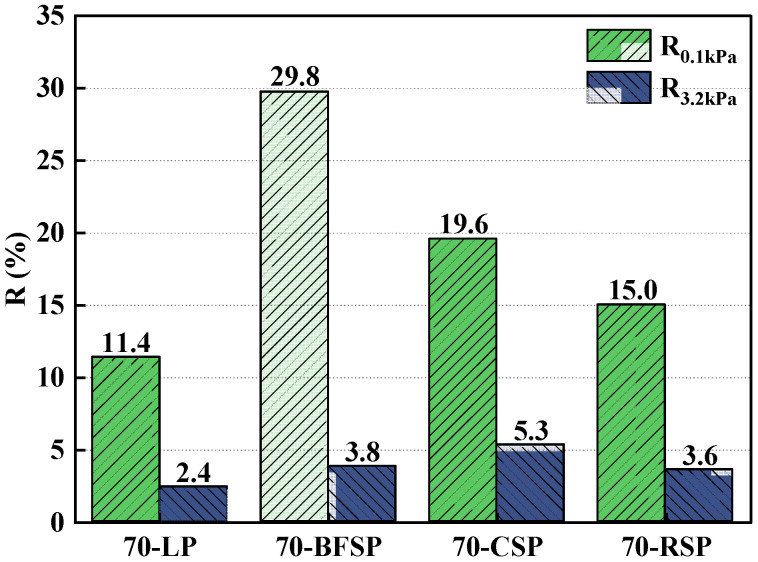
R values of asphalt mastics under the stress levels of 0.1 kPa and 3.2 kPa.

**Figure 14:**
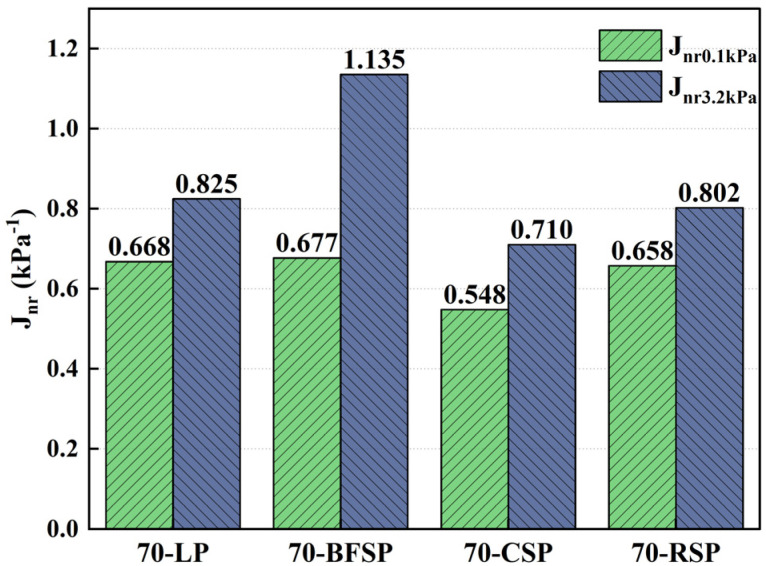
Jnr values of asphalt mastics under the stress levels of 0.1 kPa and 3.2 kPa.

**Figure 15:**
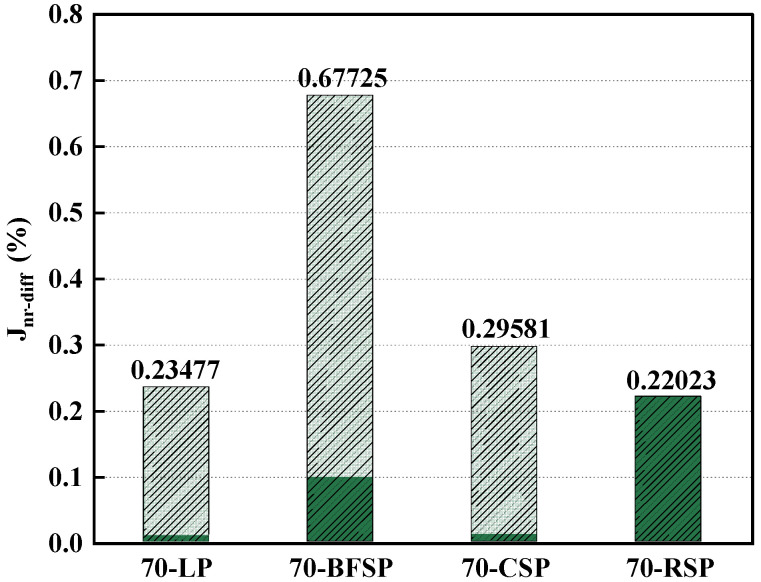
Jnr-diff values of asphalt mastics.

Based on the MSCR test, the creep recovery rate (R), the unrecoverable creep flexibility (J_nr_), and the difference rate of unrecoverable creep flexibility (J_nr-diff_) are also frequently used parameters evaluating the deformation resistance. The R reflects the resistance to elastic deformation capacity of asphalt mastic. The Jnr represents the cumulative strain of asphalt mastic. The Jnr-diff can respond to the stress sensitivity of asphalt mastic. Figure 13 and Figure 14 depict the R and Jnr of asphalt mastics under the stress levels of 0.1 kPa and 3.2 kPa. As can be seen, both 70-CSP and 70-RSP have higher R and lower Jnr than 70-LP, indicating that CSP and RSP can improve the permanent deformation resistance of asphalt mastic. Both Jnr and R of 70-BFSP are larger than that of 70-LP, indicating that BFSP does not have a negative effect on the permanent deformation resistance of asphalt mastic. Figure 15 depicts the Jnr-diff of asphalt mastics under the stress levels of 0.1 kPa and 3.2 kPa. It can be seen that the Jnr-diff values of asphalt mastics follow the order of 70-BFSP > 70-CSP > 70-LP > 70-RSP. This indicates that 70-BFSP and 70-CSP have higher sensitivity to stress compared with 70-LP and 70-RSP, which may result in the greater deformation of 70-BFSP and 70-CSP under the repeated loading. In summary, BFSP, CSP, and RSP can improve the elasticity of asphalt mastic. CSP and RSP have a good inhibition effect on the permanent deformation of asphalt mastic. BFSP results in the higher sensitivity of asphalt mastic to stress, which has an influence on the resistance to the permanent deformation of asphalt mastic.

### 5.3. Low-Temperature Crack Resistance

**Figure 16:**
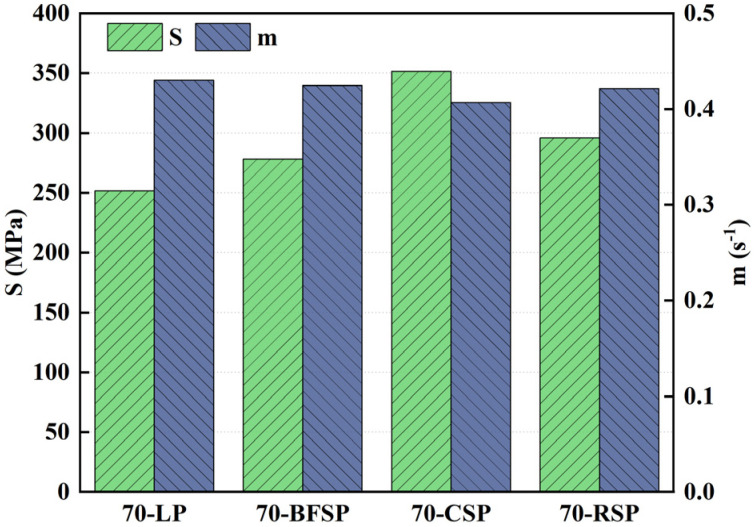
Creep strength and creep rate of each asphalt mastic.

In the BBR test, the creep strength (S) and creep rate (m) are used to evaluate the low-temperature performance. The asphalt mastic with lower S and higher m has better low-temperature cracking resistance. Figure 16 shows the results of S and m values obtained from the BBR tests. As can be seen, 70-LP has the smallest creep strength and the largest creep rate in all asphalt mastics, indicating that the steel slag powders have an adverse effect on the low-temperature cracking resistance of asphalt mastic. Moreover, 70-CSP has the largest creep strength and the smallest creep rate, indicating it has the biggest influence on the low-temperature cracking resistance of asphalt mastic. The differences in the creep strength and creep rate between BFSP and LP are small, which illustrates that BFSP has little influence on the low-temperature cracking resistance of asphalt mastic.

In summary, steel slag powder can effectively improve the microwave sensitivity property of asphalt mastic, especially RSP and CSP, and it has little influence on the viscoelasticity and anti-deformation performance of asphalt mastic. Although the low-temperature performance of asphalt mastic with steel slag filler is inferior to that of 70-LP, its creep strength and creep rate are within a reasonable range. Therefore, it is feasible to use steel slag powder as filler for enhancing the microwave sensitivity of asphalt mastic.

## 6. Microscopic Mechanism of Steel Slag Powder Impacting Asphalt Mastic Performance

**Figure 17:**
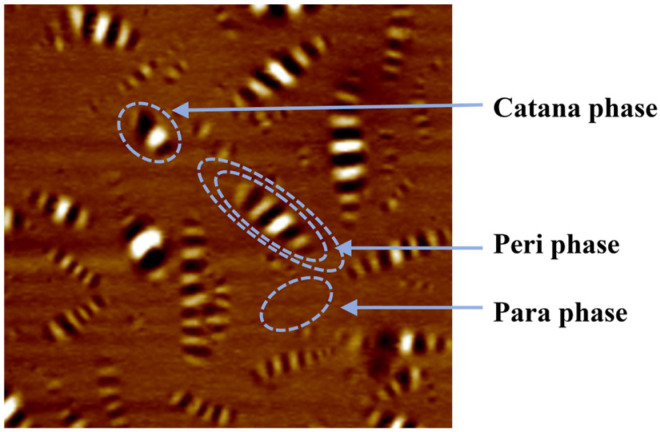
Schematic diagram of three phases of AFM image of asphalt mastic.

Figure 17 presents the schematic diagram of three phases of AFM images of asphalt mastics. As shown in this figure, the catana phase is a bee-like structure, the peri phase is the peripheral area of the bee-like structure, and the para phase is the adjacent area of the peri phase [29]. The para phase is the softest microdomain [30]. The more the para phase, the better the low-temperature performance. The bee-like structure represents the four components of asphalt. Asphalt mastic with high asphaltene and resin content has more bee-like structures. In the catana phase, the protruding white part is asphaltene, the yellow part is resins, the yellowish-brown part is aromatic, and the concave black part is saturate. The more the bee-like structures, the better the agglomeration degree of asphalt molecules, and thus the better the high-temperature performance.

**Figure 18:**
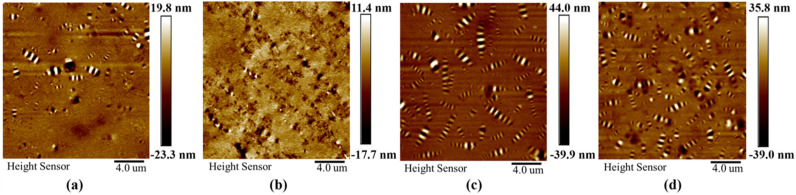
AFM images of asphalt mastic: (**a**) 70-LP, (**b**) 70-BFSP, (**c**) 70-CSP, and (**d**) 70-RSP.

Figure 18 depicts the AFM images of 70-LP, TP-BFSP, 70-CSP, and 70-RSP. As can be seen, steel slag powders have a significant influence on the micromorphology of asphalt mastics. Furthermore, 70-LP and 70-BFSP have more para phase and fewer bee-like structures compared with 70-CSP and 70-RSP. This is the reason why they have better low-temperature performance and worse anti-deformation performance.

The surface roughness index allows quantitative analysis for the information of the surface structure [31]. In this study, the mean roughness index (Ra) and the root mean square roughness index (Rq) are employed to evaluate the surface roughness of each asphalt mastic, which are calculated by Nanoscope Analysis software 3.00, according to Equations (2) and (3).

(2) $$R_{a}=\frac{\sum \left(Z_{i}\right)}{N}$$

(3) $$R_{q}=\sqrt{\frac{\sum {\left(Z_{i}\right)}^{2}}{N}}$$

where *N* is the number of selected data points within the range, and *Z_i_* is the height measurement of a surface point relative to the microscope’s operational state.

**Figure 19:**
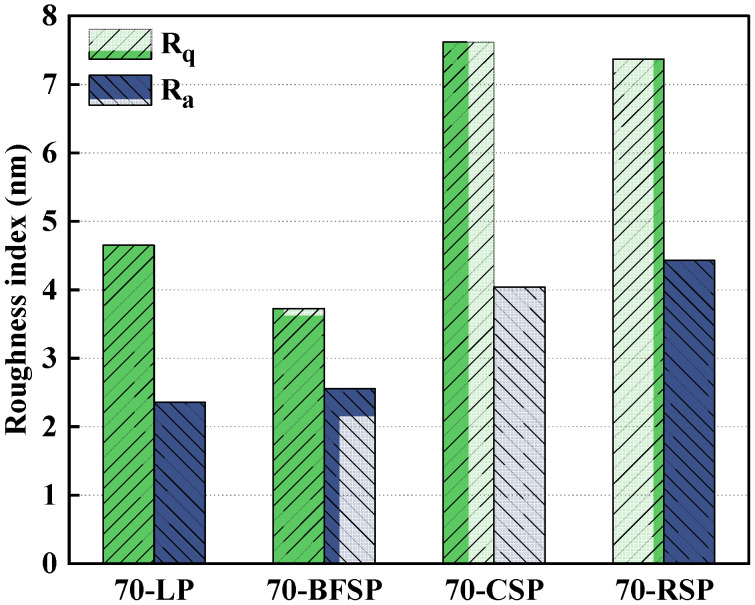
The surface roughness indexes of asphalt mastics.

Figure 19 shows the results of two surface roughness indexes of 70-LP, 70-BFSP, 70-CSP, and 70-RSP. It can be seen that 70-CSP and 70-RSP have larger surface roughness compared with 70-LP and 70-BFSP. This indicates that 70-CSP and 70-RSP have better viscosity.

## 7. Conclusions

This study analyzes the chemical composition of steel slag and limestone powders by XRD and XRF tests, evaluates the microwave absorption ability of steel slag and limestone powders by electromagnetic performance tests, compares the microwave sensitivity and performance of the asphalt mastics with different steel slag powders and LP, and reveals the effect mechanism of steel slag powder on asphalt mastic performance. The main conclusions are as follows.

(1) The chemical compositions of BFSP, CSP, RSP, and LP are similar. RSP has the strongest ability to convert microwave energy into thermal energy. CSP has a good dielectric loss ability, but its magnetic loss ability is poor. Both LP and BFSP have worse dielectric and magnetic loss properties. However, the dielectric loss ability of BFSP is higher than that of LP in the low-frequency range.
(2) Steel slag powder can improve the microwave sensitivity of asphalt mastic, especially RSP and CSP. There is a good linear relationship between the electromagnetic property of fillers and the microwave sensitivity of corresponding asphalt mastics.
(3) BFSP and RSP have little influence on the stiffness of asphalt mastic, and CSP improves the stiffness of asphalt mastic. RSP would not destroy the elastic properties of asphalt mastic, while BFSP and CSP can improve the elastic properties of asphalt mastic.
(4) Steel slag powders have an adverse effect on the low-temperature cracking resistance of asphalt mastic, but the creep strength and creep rate of asphalt mastic with steel slag powder are within a reasonable range.

In summary, steel slag powder has great potential in improving the microwave sensitivity of asphalt mastic, which is of great significance in reducing environmental pollution and promoting the application of microwave heating technology in asphalt pavement maintenance. In addition, there are significant differences in the microwave sensitivity and performance between the asphalt mastics with different steel slag powders. Therefore, this study is of guiding significance for selecting suitable steel slag according to different road service conditions.

## Figures and Tables

**Table 1:** Technical properties of steel slag and limestone fillers.

Technical Indexes	BFSP	CSP	RSP	LP
Apparent density (g/cm^3^)	3.023	3.203	3.651	2.807
Hydrophilic coefficient	0.85	1.06	0.74	0.81
Heat invariability	No color change			

**Table 2:** Technical indicators of Xinhai 70# asphalt.

Technical Indexes	Unit	70# Asphalt
Penetration (25 °C, 5 s, 100 g)	0.1 mm	67
Penetration Index (PI)	/	−0.37
Ductility (10 °C)	cm	34
Ductility (15 °C)	cm	>100
Softening Point (TR&B)	°C	48.0
Solubility	%	99.84
Flash Point	°C	268
Density (15 °C)	g/cm^3^	1.036

**Table 3:** Mass ratio of each steel slag filler to asphalt in asphalt mastic.

Fillers	LP	BFSP	CSP	RSP
Mass ratio	1.000:1	1.077:1	1.141:1	1.301:1

**Table 4:** Chemical compositions of different powders.

Filler Type	CaO	MgO	SiO_2_	Al_2_O_3_	Fe_2_O_3_	MnO
LP	59.77%	17.87%	14.85%	4.34%	1.43%	0.06%
BFSP	32.12%	11.91%	33.18%	18.64%	0.57%	0.41%
CSP	58.21%	2.43%	2.67%	30.75%	0.94%	0.07%
RSP	38.82%	6.02%	14.88%	2.85%	26.01%	5.20%

**Table 5:** The fitting results of heating curves.

Asphalt Mastics	Fitting Equation	R^2^
70-LP	*y* = 0.043*x* + 24.54	0.9898
70-BFSP	*y* = 0.137*x* + 30.38	0.9820
70-CSP	*y* = 0.336*x* + 32.52	0.9925
70-RSP	*y* = 0.441*x* + 44.35	0.9628

## Data Availability

The original contributions presented in this study are included in the article. Further inquiries can be directed to the corresponding author.

## Abbreviations

The following abbreviations are used in this manuscript:

BFSP	blast furnace slag powder
CSP	converter slag powder
RSP	refined slag powder
LP	limestone powder
XRD	X-ray diffractometer
XRF	X-ray fluorescence
AFM	atomic force microscope
DSR	dynamic shear rheometer
BBR	bending beam rheometer
BFS	blast furnace slag
CS	converter slag
RS	refined slag
MSCR	the multiple stress creep recovery
70-LP	asphalt mastic with limestone powder
70-BFSP	asphalt mastic with blast furnace slag powder
70-CSP	asphalt mastic with converter slag powder
70-RSP	asphalt mastic with refined slag powder
S	the creep strength
m	the creep rate (m)
